# A dataset on African bats’ functional traits

**DOI:** 10.1038/s41597-023-02472-w

**Published:** 2023-09-14

**Authors:** Francesca Cosentino, Giorgia Castiello, Luigi Maiorano

**Affiliations:** 1 Department of Biology and Biotechnologies “Charles Darwin”, Sapienza University of Rome, Rome, Italy; 2 Present Address: CREA Research Centre for Forestry and Wood, v.le Santa Margherita 80, 52100 Arezzo, Italy

**Keywords:** Macroecology, Ecological modelling, Biodiversity

## Abstract

Trait-based approaches are becoming extremely common in ecological modeling and the availability of traits databases is increasing. However, data availability is often biased towards particular regions and taxa, with many taxa (e.g., bats) often under-represented. Here, we present the AfroBaT dataset, a compilation of trait data on 320 African bat species containing 76,914 values for 86 traits focusing on morphology, reproduction, life-history, trophic ecology, and species distributions. All data were gathered from published literature following the ecological trait-data standard procedure. Missing data for both numerical and categorical traits were imputed with a machine learning approach including species phylogeny. Trophic ecology traits showed the highest coverage in the literature (72% of the species averaged over all traits), while reproductive traits the lowest. Our data imputation improved the coverage of AfroBaT especially for reproductive traits, going from 27% to 58% of the species covered. AfroBaT has a range of potential applications in macroecology and community ecology, and the availability of open-access data on African bats will enable collaboration and data-sharing among researchers.

## Background & Summary

The vulnerability of species to global changes depends on the intensity of changes to which they are exposed and on their intrinsic capacity to respond to these changes, mediated by their ecological traits^1^. Trait-based approaches, therefore, offer the potential to link environmental changes to biodiversity and its related functions. In this context, a trait is any feature (morphological, physiological, or phenological) that can be measured at the individual level, irrespective of the environment or other level of organization^2^.

Several databases on plant and animal traits have been published in recent years (e.g.^3,4^) with a particular focus on vertebrates (e.g.^5–7^). Mammals are very well represented (e.g., PanTHERIA^8^ COMBINE^9^), although data availability is still limited, especially for particular regions and taxa^10,11^. Bats (order Chiroptera) are under-represented in all global databases, and the regional databases focusing on bats that are available are biased towards the Neotropics and Europe^12–14^ (Table S1 in Supplementary Information).

Bats are one of the most widely distributed groups of terrestrial mammals on Earth and, with almost 1400 species^15^, they represent nearly a fifth of all mammals. They are important suppliers of ecosystem services, going from pest control to pollination, and seed dispersal^16,17^. Bats are also the natural hosts of several pathogens^18,19^, with more than 200 viruses that have been isolated or detected in bats^20^. In particular, the variety of their ecological niches, and the presence of different species, along with other abiotic and anthropogenic factors, may increase the probability of zoonotic emergence^21^. In addition, global changes are progressively reducing the distance between humans and wild species, bats included, leading to a potential increase in zoonotic diseases outbreak^22^.

In this context, monitoring and understanding the ecology of bat species represent an important research topic, and trait data can provide the keystone for regional to continental scale analyses dealing with biogeography, conservation or One Health approaches. In fact, climatic and environmental changes can have considerable impacts on phenology of bats, with effects on hibernation and reproduction, besides other negative consequences such as variation in mortality, migration and their distribution patterns^23–25^. Changes in species composition, distribution, and ecology may alter both the functions bats perform within ecosystems (with potential economic losses), and the host-pathogens interactions potentially increasing the viral sharing^22^. However, information about species-specific bat traits is often limited, especially in underdeveloped countries with limited accessibility, such as Africa, which harbors a high number of bat species along with a high density of rural human populations facilitating daily exposure of humans to wild animals^26^. The only available dataset on African bats^27^ is mainly focused on traits related to dispersal ability of Sub-Saharan bats, and therefore a more comprehensive dataset of functional traits considering the entire African continent is still lacking.

To fill this knowledge gap, we developed the AfroBaT dataset collecting trait data on African bats. All existing databases on mammals and, more in general, on tetrapods that we explored have been collated using different data sources, almost always including expert knowledge, existing literature, and other databases. AfroBaT is completely based on published literature, making it possible to completely verify the data collected. AfroBaT considers all African bat species and contains 76,914 values for a total of 86 traits including morphology, reproduction, life-history, trophic interactions, and species-specific distribution maps. The public availability of this dataset will provide an opportunity to carry out analyses on macroecology, community ecology, and host-pathogen dynamics considering the entire African continent, one of the most biodiverse continents on Earth. The dataset will be updated by the authors with new species and taxonomic revision, as well as with new data on species traits becoming available thanks to future research efforts. All future updates will be available at maioranolab.com/afrobat.

## Methods

### Taxonomic and geographical coverage

AfroBaT includes 320 species belonging to 60 genera and 13 families. We followed the taxonomy available in Wilson & Mittermeier^28^ except for six new species recently described (Table S2 in Supplementary Tables; *Miniopterus nimbae, Miniopterus wilsoni, Pipistrellus simandouensis, Pseudoromicia kityoi, Pseudoromicia nyanza, Laephotis kirinyaga*) and 17 species (Vespertilionidae) for which the taxonomy has been recently updated (Table S3 in Supplementary Tables)^29–32^.

The geographical coverage of our dataset is the entire African continent including Sinai, Madagascar, and all the other off-shore African islands including Canary Islands, Cape Verde, the Gulf of Guinea islands, Comoro Islands, Seychelles, Reunion, and Mauritius (Fig. S4 in Supplementary Information).

### Trait definitions and data sources

Following the ecological trait-data standard procedure^33^, we reviewed the literature selecting the available trait databases focused on vertebrates and, in particular, mammals (e.g.^8,34^) (Table S5 in Supplementary Information). Then, we selected functional traits and their respective field names to be included in AfroBaT, ensuring a complete harmonization of our fields with the existing literature. We prepared an ontology containing the definition for all traits (Table S6 in in Supplementary Tables), and a thesaurus for selected terms (Table S7 in Supplementary Information) found in the references. While we strived to use a standardized vocabulary that allows easy comparisons with other trait ontologies, we formulated our own ontology for traits not available in the already existing ontology libraries collected in the Ontobee Data Server (https://ontobee.org/).

Trait data were gathered from Wilson & Mittermeier, Monadjem *et al*., and Kingdon^28,35,36^. For all traits related to specific trophic items (‘AfroBaT_detailed_diet’) we used the Bat Eco-Interactions database (https://www.batbase.org/) which includes visitation, consumption, and predation of food resources.

For all missing data, we used a machine learning data imputation approach for both numeric and categorical traits including phylogeny^37,38^. We explicitly distinguished all imputed data from the data available in the literature by providing two separated datasets, the original dataset and the imputed dataset.

**Fig. 1:**
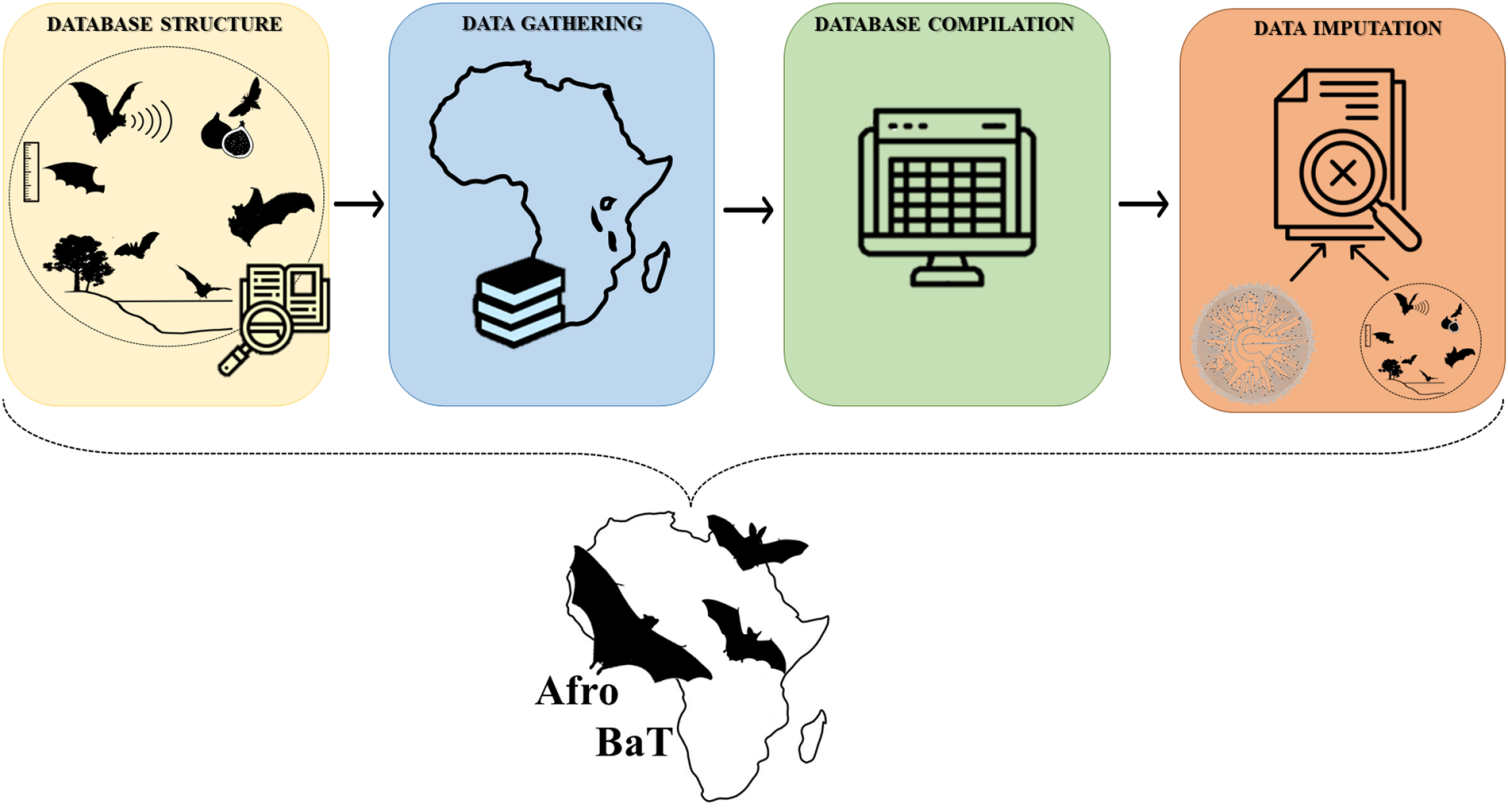
Graphical workflow of AfroBaT dataset construction.

The final AfroBaT dataset includes 320 bat species with a total of 86 traits, focusing on morphology (18 traits), reproduction (16 traits), life-history (20 traits), trophic guild (4 traits), feeding space (4 traits), foraging habitat (3 traits), feeding strategy (6 traits), general diet (13 items), and detailed diet (Fig. 1; Tables S8–S18 in Supplementary Tables). For each species we also collected a species distribution map from Cosentino *et al*.^39^.

### Dataset compilation protocol

We filled out the dataset by consulting the three main references selected plus the available literature for the newly described species. We used Wilson & Mittermeier (2019) as the main reference since it is currently the most complete source of data on bats. Whenever the information was missing in the species-specific section, we obtained the data from the family section only where it was made clear that the information concerned all species of the considered family. When trait values were not present in our main reference, we checked also the other two selected references.

Whenever possible, we included in AfroBaT only traits measured in African populations for each species. Where no measurement was available for Africa, we considered also traits measured outside of our study area. When data for a trait were available for more than one African population, we included in the dataset the average value. For morphology, reproduction, and life-history traits, we collected for each species and for each numeric trait, where applicable, the minimum, maximum, and average values for male, female, and unsexed individuals. Categorical traits are expressed in binary form (presence/absence) except for the traits ‘dental formula’, ‘mating system’, ‘colony size’, and ‘echolocation type’ which have specific categorical values (see Tables S8–S18 in Supplementary Tables).

A few traits were flagged automatically in the same way for all species unless we found a different indication in the literature. When no information on acoustic behavior was provided by the references for an insectivorous species, we filled out ‘Echolocation’ as present. When no information on activity pattern was available, we flagged the field ‘Activity pattern Night’ as present for all species. When evidence of supplying ecosystem services was not provided by the references, we assumed that frugivorous species provide seed dispersal and insectivorous species provide pest control.

All traits related to trophic ecology are expressed as the frequency (in categories) of a given trait or item in the diet. In particular, 0 represents the absence of a particular item in the diet or of a particular behavior in feeding, 1 represents a behavior/item that is possible but not usual for a species, and 2 represents a behavior/item that is typical for a given species.

### Imputation of missing trait data

We included in the missing data imputation procedure only traits for which at least 15% of the species have a value in AfroBaT. To limit the amount of variability in our analyses, we only imputed data for unsexed individuals; when no data was available for unsexed, we filled the dataset with the average value calculated for males and females, or with the available value when only one sex was known. We excluded from data imputation all trophic ecology traits, which are typically extremely variable even between sister species^40^. The final set of traits on which we performed data imputation included 5 morphology, 9 reproduction, and 5 life-history traits (Table S19 in Supplementary Information).

To increase the reliability of our data imputation procedure^37^, we included bat phylogeny from VertLife^41^ as one of the predictor variables. We downloaded 100 phylogenetic node-based trees, and we calculated the phylogenetic eigenvectors through a principal coordinate analysis (PCoA; *PVR* R package, R 4.1.2). We included in the analyses the first 100 eigenvectors for each phylogenetic tree, representing 95% of the phylogenetic variance. Since the phylogenetic information was not available for all bat species (Table S20 in Supplementary Tables), we only considered 286 out of 320 bat species.

Data imputation was calibrated using the *missForest* R package, which is particularly appropriate for highly dimensional and mixed data^38^. For each of the 100 phylogenetic trees, we obtained 100 imputed datasets. The final values were calculated as the average value for numeric traits and as the mode for categorical traits. All traits with imputed values were merged with the original AfroBaT dataset.

### Species distributions

Species distribution maps represent the potential distribution of each species according to the outputs of species-specific species distribution models (SDMs) presented for the first time in the AfroBaT dataset. Methods of the species-specific SDMs follow Cosentino *et al*. (2023). While, for species with less than 20 occurrences, we calibrated at 1 km^2^ resolution a bioclimatic envelope model^42^ which considers the same variables as the models obtained from Cosentino *et al*.^39^.

## Data Records

### Access

AfroBaT dataset is stored and available for download at figshare data repository^43^ with all future updates that will be available at maioranolab.com/afrobat. The dataset is organized in four folders: the folders ‘AfroBaT pre-imputation dataset’ and ‘AfroBaT final dataset’ contain 11 csv files each, pre- and post-imputation. These files represent one table for each type of trait described above. The third folder (‘AfroBaT imputation procedure’) contains all files required for the data imputation procedure (sub-folder with phylogenetic data, subset of traits, and R script). Each folder contains a ‘read_me.txt’ file with the data descriptor. The species distribution maps are stored in the folder ‘AfroBaT SDMs’ which contains six NetCDF files (from file ‘sp1.nc’ to ‘sp6.nc’) representing the species-specific SDMs of 292 out of 320 species in alphabetic order (Tables S21, S22 in Supplementary Tables).

### Data coverage

#### Pre-imputation data coverage

AfroBaT includes 86 traits for each of the 320 species (13 bat families), summing up to 76,914 values. Trophic ecology traits were the ones with the highest species coverage, while reproductive traits were the least represented in the literature selected. In particular, trophic ecology traits were available for almost 72% of the species averaged over all trophic ecology traits, with ‘trophic guild’ and ‘general diet’ being available for all species, while ‘detailed diet arachnids’ and ‘foraging habitat’ were available for 47% of the species. Reproductive traits were available for 27% of the species. On one hand, ‘Age of first birth mean’ was filled only for 8 species, while on the opposite ‘Litter size minimum’ was available for 100% of the species (being always 1 the minimum litter size), followed by ‘Active gestation length mean’ with information for 43% of the species. Morphological traits were available for almost 48% of the species averaged over all morphological traits, with ‘Sexual Dimorphism’, ‘Dental Formula’, and ‘Number of teeth’ known for almost all species (from 98% to 100% of the species). Life-history traits were available for 54% of the species averaged over all traits, with ‘Echolocation’, ‘Activity pattern’, ‘Conservation status’, and all ecosystem services traits covered for 100% of the species, while ‘Dispersal distance’ being completely unknown (0% of the species).

#### Post-imputation data coverage

The data imputation procedure produced reliable results, with an average NRMSE (Normalized Root Mean Squared Error) of 1.46 for numeric variables, and an average PCF (Proportion of Falsely Classified) of 0.14 for categorical ones. The data coverage with data imputation increased to 31% of the species for reproductive traits, reaching a total of 58% of the species covered on average considering all reproductive traits. The improvement for morphological and life-history traits was more limited, resulting in a data coverage of 49% and 62% of the species respectively.

## Technical Validation

To test the reliability of our compilation, a random subsample with 10% of the species was compiled independently by two authors (F.C. and G.C.) given the same ontology and the same thesaurus. We measured the disagreement between the authors, and we found that only 0.4% of the values were different due to misinterpretation of the references. In a further 2.4% of the values, one of the two compilers filled out the trait with ‘NA’ (Not Available) instead of the available value overlooking a trait value that was actually present in the references.

### Supplementary information

Supplementary Information

Supplementary Tables

## Data Availability

The script for the pre- and post-data imputation processing was developed in R 4.1.2, and it is available at Figshare (folder ‘AfroBaT imputation procedure^43^).
