# Supplementary material for: A dataset on African bats’ functional traits
Source: Sci Data. 2023 Sep 14;10:623. doi: 10.1038/s41597-023-02472-w (PMC10502069; doi:10.1038/s41597-023-02472-w)
Supplement: Supplementary file 1 — Supplementary Information [file 41597_2023_2472_MOESM1_ESM.pdf]

SUPPLEMENTARY INFORMATION

A dataset on African bats' functional traits

Francesca Cosentino[1]*, Giorgia Castiello[1,§], Luigi Maiorano[1]

[1.] Department of Biology and Biotechnologies "Charles Darwin", Sapienza University of Rome

*Corresponding author: francesca.cosentino@uniroma1.it

§ current affiliation: CREA Research Centre for Forestry and Wood, v.le Santa Margherita 80, 52100 Arezzo, Italy

SUPPLEMENTARY INFORMATION CAPTIONS

- Table S1. List of currently available database on Chiroptera.
- *Table S2. List of African bats species described from 2019 to 2021.
- *Table S3. List of African bats species for which the taxonomy has been updated in 2020.
- Figure S4. Geographical coverage of the AfroBaT dataset.
- Table S5. List of vertebrate trait databases used to select trait and field names in AfroBaT.
- *Table S6. Traits ontology definitions with cross-references. When definitions were not available in other trait ontologies, we built our own ontology.
- Table S7. Thesaurus of terminology used in AfroBaT, and the equivalent expressions found in the references used.
- *Table S8. Morphological traits reported in AfroBaT. Minimum, maximum, and average values were collected for each numeric variable except for the ‘Number of teeth’ which has a single, normal value for each species. In ‘Dental formula’ the types of teeth are represented by the first letter (I= Incisor; C=Canine; P=Premolar; M=Molar) followed by the number of teeth of the upper and the lower jaw respectively separated by a dash. F/M/U indicates variables collected for female (F), male (M), and unsexed (U) individuals. N/a = Not applicable. A description of each trait is given in the ontology table (Table S6). Data are available in “AfroBaT_morphology.csv”.
- *Table S9. Reproductive traits reported in AfroBaT. Minimum, maximum, and average values were collected for each numeric variable. ‘Number of litters per year’ included only average and maximum (the minimum would always be 0 for all species, indicating a failure in reproduction). ‘Interval between births’ included only minimum and average (the maximum having no biological sense in case of reproductive failure). F/M/U indicates variables collected for female (F), male (M), and unsexed (U) individuals. N/a = Not applicable. A description of each trait is given in the ontology table (Table S6). Data are available in “AfroBaT_reproduction.csv”.
- *Table S10. Life-history traits reported in AfroBaT. Minimum, maximum, and average values were collected for each numeric variable. F/M/U indicates variables collected for female (F), male (M), and unsexed (U) individuals. N/a = Not applicable. A description of each trait is given in the ontology table (Table S6). Data are available in “AfroBaT_life_history.csv”.
- *Table S11. Trophic guild traits reported in AfroBaT. N/a = Not applicable. A description of each trait is given in the ontology table (Table S6). Data are available in “AfroBaT_trophic_guild.csv”.
- *Table S12. Feeding space traits reported in AfroBaT. N/a = Not applicable. A description of each trait is given in the ontology table (Table S6). Data are available in “AfroBaT_feeding_space.csv”.
- *Table S13. Foraging habitat traits reported in AfroBaT. N/a = Not applicable. A description of each trait is given in the ontology table (Table S6). Data are available in “AfroBaT_foraging_habitat.csv”.

- *Table S14. Feeding strategy traits reported in AfroBaT. N/a = Not applicable. A description of each trait is given in the ontology table (Table S6). Data are available in “AfroBaT_feeding_strategy.csv”.
- *Table S15. General diet traits items reported in AfroBaT. N/a = Not applicable. A description of each trait is given in the ontology table (Table S6). Data are available in “AfroBaT_general_diet.csv”.
- *Table S16. List of plant families and genus considered in the ‘AfroBaT_detailed_diet_plants’ table. The lower taxonomic level is given only if mentioned in the reference.
- *Table S17. List of insect orders and families considered in the ‘AfroBaT_detailed_diet_insects’ table. The lower taxonomic level is given only if mentioned in the reference.
- *Table S18. List of arachnid orders and families considered in the ‘AfroBaT_detailed_diet_arachnids’ table. The lower taxonomic level is given only if mentioned in the reference.
- Table S19. Subset of traits considered for the data imputation.
- *Table S20. List of African bats considered in AfroBaT dataset with information on the availability of phylogenetic data (1=yes; 0=no) and related synonyms in VertLife database (if present). In the column ‘Nomenclature in trait datasets’ are reported the species for which we have updated the nomenclature in the trait datasets.
- *Table S21. List of files within AfroBaT dataset.
- *Table S22. List of species-specific SDMs within each NetCDF file. Species with name “_avg.tif” represent SDMs from Cosentino et al. (2023); species with name “_bc.tif” represent bioclim models.

*Tables with asterisk are provided separately in the Excel file ‘Supplementary Tables.xlsx’.

Table S1. List of currently available database on Chiroptera.

**Database**	**Description**	**Reference**
A database of common vampire bat reports	Database of common vampire bat historical occurrence reports in more than 39,000 localities across the Americas.	Van de Vuurst *et al*. A database of common vampire bat reports. *Scientific data* **9**, 1-7 (2022). https://doi.org/10.6084/m9.figshare.15025296
Bat eco-interactions database	Database of interactions of bats with other organisms worldwide including the taxonomic information, the type of interaction (pollination, visitation, consumption, host, etc.) and the details of the location.	Geiselman, C. K., & Younger, S. (2020). Bat Eco-Interactions Database. https://www.batbase.org/
BGD	Database of bat genomes and genes from six bat species, including two megabats and four microbats.	Fang, J., Wang, X., Mu, S., Zhang, S., & Dong, D. BGD: A database of bat genomes. *PLoS One* **10** (2015). http://donglab.ecnu.edu.cn/databases/BatGenome/
DarkCideS 1.0	Database including geographical location, ecological status, species traits, and parasites and hyperparasites for 679 bat species that are known to occur in caves.	Tanalgo, K. C. *et al.* DarkCideS 1.0, a global database for bats in karsts and caves. *Scientific Data* **9**, 1-12 (2022). https://darkcides.org/
DBatVir	Database including 4,100 bat-associated viruses of 23 viral families detected from 196 bat species in 69 countries worldwide. It contains viral sequences, bat samples, sampling time, location, bat species and specimen type.	Chen, L., Liu, B., Yang, J., & Jin, Q. DBatVir: the database of bat-associated viruses. *Database* (2014). http://www.mgc.ac.cn/DBatVir/
EuroBaTrait 1.0	A species-level trait dataset of bats in Europe and beyond. It includes data for 47 European bat species on genetic composition, physiology, morphology, acoustic signature, climatic associations, foraging habitat, roost type, diet, spatial behaviour, life history, pathogens, phenology, and distribution	Froidevaux, J.S.P. *et al.* A species-level trait dataset of bats in Europe and beyond. *Scientific data* **10**, 253 (2023). https://doi.org/10.6084/m9.figshare.21777161.v2 (2023)
NeoBat Interactions	Database of interactions between 93 bat species of the family Phyllostomidae and 501 plant species of 68 families in the Neotropics.	Florez-Montero, G. L. *et al.* NeoBat Interactions: A data set of bat–plant interactions in the Neotropics (2022). https://zenodo.org/record/4894176

*Table S2. List of African bats species described from 2019 to 2021.

*Table S3. List of African bats species for which the taxonomy has been updated in 2020.

Figure S4. Geographical coverage of the AfroBaT dataset.

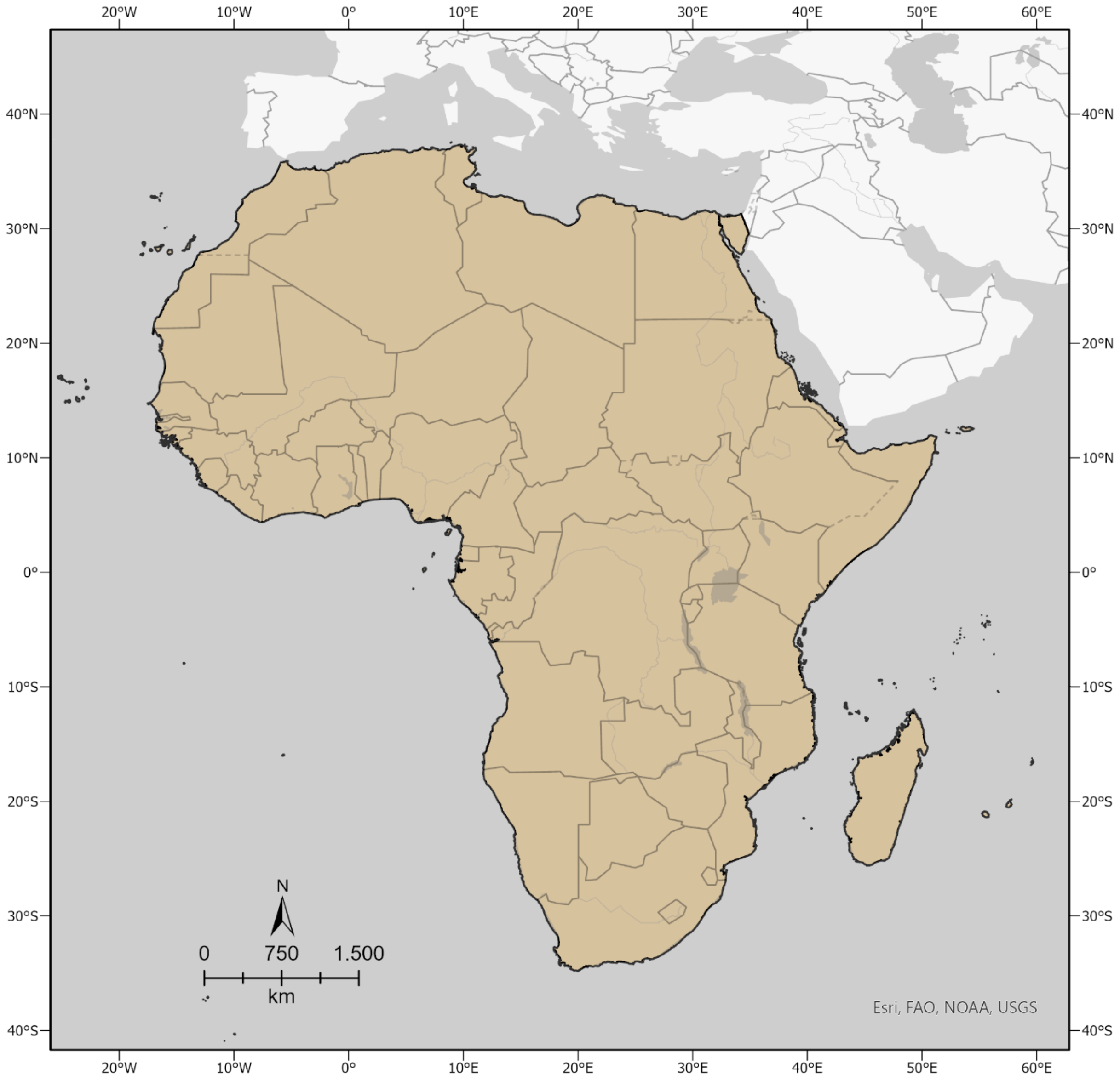

Table S5. List of vertebrate trait databases used to select trait and field names in AfroBaT.

Database	Description	Reference
Amniote Life History Database	Database containing 29 life-history parameters for 21,322 species of birds, mammals, and reptiles.	Myhrvold, N. P. *et al.* An amniote life-history database to perform comparative analyses with birds, mammals, and reptiles: Ecological Archives E096-269. *Ecology* **96**, 3109-3109 (2015). https://esapubs.org/archive/ecol/E096/269/
AnAge	Database of longevity records and life history traits for more than 4,000 vertebrate species.	De Magalhaes, J. P., & Costa, A. J. A database of vertebrate longevity records and their relation to other life-history traits. Journal of evolutionary biology **22**, 1770-1774 (2009). http://genomics.senescence.info/species/
PanTHERIA	Database of life history, ecology, and geography of extant and recently extinct global mammals including mammalian geographic ranges and global climatic and anthropogenic variables.	Jones, K. E. *et al.* PanTHERIA: a species-level database of life history, ecology, and geography of extant and recently extinct mammals: Ecological Archives E090-184. *Ecology* **90**, 2648-2648 (2009). https://tinyurl.com/5czp2t33
VertNet	Biodiversity data publishing organization, which aggregates vertebrate data published by natural history collections from all over the world using the Darwin Core standard.	Guralnick, R., & Constable, H. VertNet: creating a data-sharing community. *BioScience* **60**, 258-259 (2010). http://portal.vertnet.org/search

*Table S6. Traits ontology definitions with cross-references. When definitions were not available in other trait ontologies, we built our own ontology.

Table S7. Thesaurus of terminology used in AfroBaT, and the equivalent expressions found in the references used.

**Trait name**	**Equivalent expressions**
Mating system	Breeding season; Mating
Mating system = 3 = Polygyny	Harem
Age of first birth	Age of first reproduction
Sperm retention	Delayed fertilization
Nursery roost	Maternity roost; Maternity colony; Nursery colony
Colony size number	• Several individuals (reported as 9 individuals);<br>• Dozens/Several dozen/Tens of individuals (all reported as 99 individuals);<br>• A few hundred individuals (reported as 500 individuals);<br>• Hundreds of individuals (reported as 999 individuals)
Colony size	• 1-99 individuals (reported as small);<br>• 100-9999 individuals (reported as medium);<br>• >9999 individuals (reported as large)
Hibernation	Diapause
Frequency of maximum energy	Peak frequency
End frequency	Minimum frequency (only if FM call); Final frequency
Aerial	Aerial prey
Hawking	
Nectar	Pollen
Insects	Insectivorous; Invertebrates; Other arthropods
Arachnids	
Centipedes	
Blattodea (Termitidae)	Isoptera
Coleoptera	Beetles
Hemiptera	Bugs

*Table S8. Morphological traits reported in AfroBaT. Minimum, maximum, and average values were collected for each numeric variable except for the ‘Number of teeth’ which has a single, normal value for each species. In ‘Dental formula’ the types of teeth are represented by the first letter (I= Incisor; C=Canine; P=Premolar; M=Molar) followed by the number of teeth of the upper and the lower jaw respectively separated by a dash. F/M/U indicates variables collected for female (F), male (M), and unsexed (U) individuals. N/a = Not applicable. A description of each trait is given in the ontology table (Table S6). Data are available in “AfroBaT_morphology.csv”.

*Table S9. Reproductive traits reported in AfroBaT. Minimum, maximum, and average values were collected for each numeric variable. ‘Number of litters per year’ included only average and maximum (the minimum would always be 0 for all species, indicating a failure

in reproduction). ‘Interval between births’ included only minimum and average (the maximum having no biological sense in case of reproductive failure). F/M/U indicates variables collected for female (F), male (M), and unsexed (U) individuals. N/a = Not applicable. A description of each trait is given in the ontology table (Table S6). Data are available in “AfroBaT_reproduction.csv”.

*Table S10. Life-history traits reported in AfroBaT. Minimum, maximum, and average values were collected for each numeric variable. F/M/U indicates variables collected for female (F), male (M), and unsexed (U) individuals. N/a = Not applicable. A description of each trait is given in the ontology table (Table S6). Data are available in “AfroBaT_life_history.csv”.

*Table S11. Trophic guild traits reported in AfroBaT. N/a = Not applicable. A description of each trait is given in the ontology table (Table S6). Data are available in “AfroBaT_trophic_guild.csv”.

*Table S12. Feeding space traits reported in AfroBaT. N/a = Not applicable. A description of each trait is given in the ontology table (Table S6). Data are available in “AfroBaT_feeding_space.csv”.

*Table S13. Foraging habitat traits reported in AfroBaT. N/a = Not applicable. A description of each trait is given in the ontology table (Table S6). Data are available in “AfroBaT_foraging_habitat.csv”.

*Table S14. Feeding strategy traits reported in AfroBaT. N/a = Not applicable. A description of each trait is given in the ontology table (Table S6). Data are available in “AfroBaT_feeding_strategy.csv”.

*Table S15. General diet traits items reported in AfroBaT. N/a = Not applicable. A description of each trait is given in the ontology table (Table S6). Data are available in “AfroBaT_general_diet.csv”.

*Table S16. List of plant families and genus considered in the ‘AfroBaT_detailed_diet_plants’ table. The lower taxonomic level is given only if mentioned in the reference.

*Table S17. List of insect orders and families considered in the ‘AfroBaT_detailed_diet_insects’ table. The lower taxonomic level is given only if mentioned in the reference.

*Table S18. List of arachnid orders and families considered in the ‘AfroBaT_detailed_diet_arachnids’ table. The lower taxonomic level is given only if mentioned in the reference.

Table S19. Subset of traits considered for the data imputation.

**Trait name**	**Stats**	**Trait table**
HeadBody_Length (Unsexed)	min/max/mean	Morphology
Tail_Length (Unsexed)	min/max/mean	Morphology
Forearm_Length (Unsexed)	min/max/mean	Morphology
Hindfoot_Length (Unsexed)	min/max/mean	Morphology
Body Mass (Unsexed)	min/max/mean	Morphology
Sperm_Retention	N/a	Reproduction
Delayed_Implantation	N/a	Reproduction
Delayed_Development	N/a	Reproduction
Active_Gestation_Length	mean	Reproduction
Total_Gestation_Length	mean	Reproduction
Lactation_Length	mean	Reproduction
Litter_Size	mean	Reproduction
Num_Litter_year	mean	Reproduction
Births_Interval	mean	Reproduction
Migratory	N/a	Life-history
Colony_Size (numeric)	min/max/mean	Life-history
Colony_Size (categorical)	N/a	Life-history
Hibernation_Length	min/max/mean	Life-history
Activity_Pattern	N/a	Life-history

*Table S20. List of African bats considered in AfroBaT dataset with information on the availability of phylogenetic data (1=yes; 0=no) and related synonyms in VertLife database (if present). In the column 'Nomenclature in trait datasets' are reported the species for which we have updated the nomenclature in the trait datasets.

*Table S21. List of files within AfroBaT dataset.

*Table S22. List of species-specific SDMs within each NetCDF file. Species with name "_avg.tif" represent SDMs from Cosentino et al. (2023); species with name "_bc.tif" represent bioclim models.
